# Supplementary material for: Framework for the Simulation of Sensor Networks Aimed at Evaluating In Situ Calibration Algorithms
Source: Sensors (Basel). 2020 Aug 14;20(16):4577. doi: 10.3390/s20164577 (PMC7472635; doi:10.3390/s20164577)
Supplement: Supplementary file 1 [file sensors-20-04577-s001.pdf]

Delaine, F.; Lebental, B.; Rivano, H. Example case study applying a "Framework for the Simulation of Sensor Networks Aimed at Evaluating In Situ Calibration Algorithms". Université Gustave Eiffel, **2020**. doi:10.25578/CJCYMZ.
